# B-Type Natriuretic Peptide as a Significant Brain Biomarker for Stroke Triaging Using a Bedside Point-of-Care Monitoring Biosensor

**DOI:** 10.3390/bios10090107

**Published:** 2020-08-26

**Authors:** Dorin Harpaz, Raymond C. S. Seet, Robert S. Marks, Alfred I. Y. Tok

**Affiliations:** 1 School of Material Science & Engineering, Nanyang Technology University, 50 Nanyang Avenue, Singapore 639798, Singapore; miytok@ntu.edu.sg; 2 Department of Biotechnology Engineering, Ben-Gurion University of the Negev, Beer-Sheva 84105, Israel; rsmarks@bgu.ac.il; 3 Division of Neurology, Department of Medicine, Yong Loo Lin School of Medicine, National University of Singapore, NUHS Tower Block, 1E Kent Ridge Road, Singapore 119228, Singapore; mdcrscs@nus.edu.sg

**Keywords:** stroke, diagnostics, biomarker, b-type natriuretic peptide, point-of-care, biosensor

## Abstract

Stroke is a widespread condition that causes 7 million deaths globally. Survivors suffer from a range of disabilities that affect their everyday life. It is a complex condition and there is a need to monitor the different signals that are associated with it. Stroke patients need to be rapidly diagnosed in the emergency department in order to allow the admission of the time-limited treatment of tissue plasminogen activator (tPA). Stroke diagnostics show the use of sophisticated technologies; however, they still contain limitations. The hidden information and technological advancements behind the utilization of biomarkers for stroke triaging are significant. Stroke biomarkers can revolutionize the way stroke patients are diagnosed, monitored, and how they recover. Different biomarkers indicate different cascades and exhibit unique expression patterns which are connected to certain pathologies in the human body. Over the past decades, B-type natriuretic peptide (BNP) and its derivative N-terminal fragment (NT-proBNP) have been increasingly investigated and highlighted as significant cardiovascular biomarkers. This work reviews the recent studies that have reported on the usefulness of BNP and NT-proBNP for stroke triaging. Their classification association is also presented, with increased mortality in stroke, correlation with cardioembolic stroke, and an indication of a second stroke recurrence. Moreover, recent scientific efforts conducted for the technological advancement of a bedside point-of-care (POC) device for BNP and NT-proBNP measurements are discussed. The conclusions presented in this review may hopefully assist in the major efforts that are currently being conducted in order to improve the care of stroke patients.

## 1. Introduction

Stroke is a leading cause of death that causes 7 million deaths globally [1]. It occurs as a result of an acute interruption in the brain blood flow [2]. This interference is either due to ischemic stroke (blockage) in 87% of cases, or due to haemorrhagic stroke (bleeding) in the remaining 13% of cases [3,4]. Stroke patients suffer from a range of disabilities that affect their everyday life [1,5]. Stroke is a complex condition and there is a need to monitor the different signals that are associated with it [6]. For example, in the 90 days prior to the stroke event, patients usually suffer from transient ischemic attacks (TIAs) [7]. In this period, there are significant approaches that can be used in order to reduce the risk of stroke [8]. The most common causes for stroke and TIA are embolic or thrombotic consequences of atherothrombotic disease [9]. They share several risk factors, including dietary factors, physical inactivity, excessive alcohol intake, obesity, diabetes, smoking, dyslipidaemia, and hypertension [10]. Individuals of any age can suffer from a stroke; however, it is most common in the elderly population (>65 years) [11,12]. Brain changes related to aging have been linked to stroke; moreover, stroke mechanisms differ between young and elderly patients [13]. Together with the growth of the aging population, stroke prevalence and costs will significantly rise in the following years [14,15,16]. A stroke victim is also considered to be in an increased health risk for heart function, breathing abnormalities, body temperature control, and paralysis [6]. Stroke patient recovery ranges from months at best but can take years, which increases the burden and costs on the healthcare system in developed countries worldwide [14,15,16].

The identification of patients with acute stroke in the emergency department (ED) is necessary [17] in order to deliver therapies with time-limited treatment, including intravenous and intra-arterial tissue plasminogen activator (tPA) [18,19,20,21,22,23]. Brain cells die rapidly after stroke; therefore, therapeutic treatment needs to be administered early. The ‘time–outcome’ effect is highly important in stroke monitoring [24,25,26,27] due to the association of time delays with worse patient outcomes [28,29,30]. The most common modality for evaluation of suspected stroke patients is magnetic resonance imaging (MRI) or computed tomography (CT), which are mainly used to exclude intra-cerebral hemorrhage but have poor sensitivity for detecting acute ischemia. Limitations also include availability, costs, and radiation exposure [4,31,32]. Stroke diagnostics show the use of sophisticated technologies; however, they still contain limitations [33]. The widely used stroke scoring systems, such as the National Institutes of Health Stroke Scale (NIHSS) [34,35], are not able to distinguish patients with acute stroke from those with mimic presentations, such as metabolic disorders, migraines, and mass brain lesions [36]. Poor stroke diagnostics results in both pre-hospital and ED time delays in stroke care [37]. Approximately 30% of patients that were initially suspected to have a stroke are eventually diagnosed with an unrelated condition [38]. Moreover, 50% of stroke cases are missed [39,40,41,42]. A second priority for stroke patients’ care is to improve the patient’s recovery after stroke. There are approximately 30 million stroke survivors globally, and they comprise approximately one-quarter of the residents in long-term care (LTC) facilities. The care for stroke survivors in LTC facilities is lacking in rehabilitation, stroke-specific care, and secondary stroke prevention [43]. Additionally, it is necessary to continue in follow-up examinations, which are essential for coordinating post-acute services and monitoring of the patient’s progress [44]. In order to improve the acute ischemic stroke diagnostic, a potential strategy is to measure the serum levels of brain-injury-related biochemical biomarkers [45,46]. In addition, there is a need for an improved recovery approach and the use of biomarkers has promising results [47].

**Figure 1:**
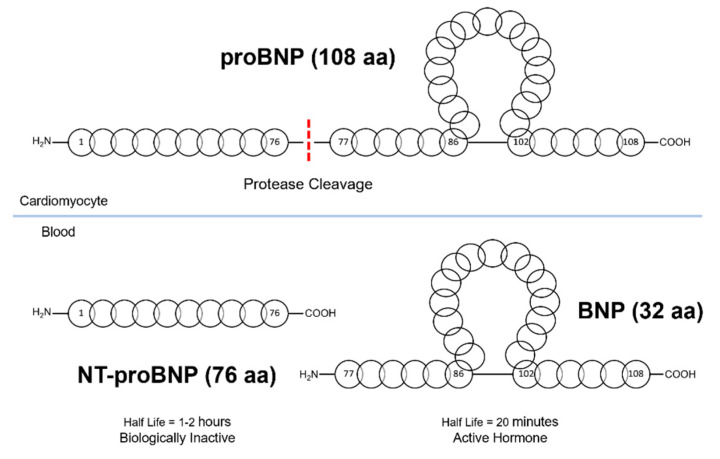
The biochemistry of B-Type natriuretic peptide expression.

**Figure 2:**
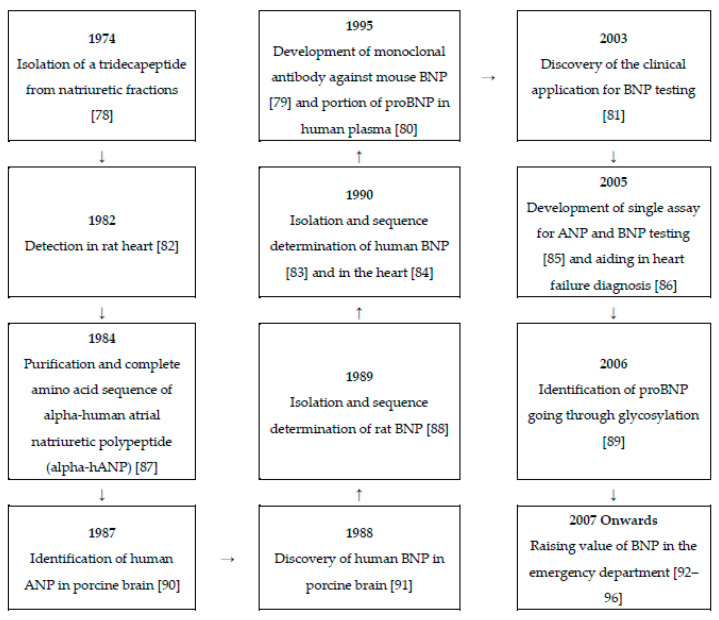
Timeline of the research developments on natriuretic peptides [78,79,80,81,82,83,84,85,86,87,88,89,90,91,92,93,94,95,96].

The hidden information and technological advancements behind the utilization of biomarkers for stroke triaging are significant [48,49,50]. Stroke biomarkers can revolutionize the way that stroke patients are diagnosed, monitored, and how they recover [51,52,53]. Different biomarkers indicate different cascades and exhibit unique expression patterns that are connected to certain pathologies in the human body [46,54,55]. A biomarker should be specific (reflect the extent of brain damage), sensitive (easily detected), and selective (reflect therapeutic efficacy) [56,57]. Stroke is associated with a variety of pathophysiological changes, which lead to the triggering of different biochemical processes and related biomarkers [58,59,60,61,62,63,64,65,66,67,68,69,70]. Over the past decades, B-type natriuretic peptide (BNP) and its derivative N-terminal pro-BNP (NT-proBNP) have been increasingly investigated and highlighted as significant cardiovascular biomarkers, especially for heart failure (HF) and recently also for stroke [71,72]. The biochemistry of BNP expression is presented in Figure 1. BNP is also referred to as uretic peptide or ventricular natriuretic peptide. It is a 32-amino acid long cyclic polypeptide with the ring structure confined between cysteine in positions 10 and 26. It is secreted by the heart ventricles as a result of excessive stretching of cardiomyocytes (heart muscle cells) [73]. Although BNP was discovered in porcine brain, subsequent studies revealed that in humans, BNP is most abundantly expressed in the heart [74]. Recent studies have shown that proBNP is glycosylated, which results in the inhibition of processing of proBNP by protease (e.g., furin or corin). Therefore, the concentration of proBNP in the blood is higher than of BNP [75]. BNP release is also regulated by calcium ions. The biologically active BNP is secreted together with the biologically inactive 76-amino acid NT-proBNP peptide. Similarly to atrial natriuretic peptide (ANP), BNP binds and activates the atrial natriuretic peptide receptors NPRA, but with 10-fold lower affinity [76]. However, the biological half-life of ANP is half of that of BNP, and NT-proBNP has an even longer half-life time, making both BNP and NT-proBNP better candidates for a diagnostic device. BNP accurately reflects current ventricular status, as its half-life is 20 min, as opposed to 1–2 h for NT-proBNP [77]. The timeline of the research developments on natriuretic peptides is presented in Figure 2. This work reviews the recent studies that have reported on the usefulness of BNP and NT-proBNP for stroke triaging. Then, their association is presented with increased mortality in stroke, correlation with cardioembolic stroke, and an indication of second stroke recurrence. Moreover, the recent scientific efforts conducted for the technological advancements in a bedside point-of-care (POC) device for BNP and NT-proBNP measurements are discussed.

## 2. B-Type Natriuretic Peptide Expression in Stroke Pathology

### 2.1. Association with Increased Mortality in Stroke

BNP plasma and NT-proBNP serum levels are elevated in stroke pathology (Table 1) and associated with increased mortality [97,98,99,100,101,102,103,104,105,106,107,108]. Montaner J. et al. [104] identified that BNP plasma levels were higher among patients who died (118.2 vs. 60.9 pg/mL; *p* < 0.001), and multivariate logistic regression analysis indicated that the plasma BNP level was an independent predictor of death after stroke (BNP > 65.3 pg/mL; odds ratio (OR) = 1.97; *p* = 0.034). Chen X. et al. [103] reported that NT-proBNP levels in the deceased group (3280 pg/mL) were significantly (*p* < 0.001) higher than in the survival group (926.30 pg/mL). García-Berrocoso T. et al. [97] concluded that BNP is associated with post-stroke mortality independent of the NIH stroke scale score, age, and sex. However, their translation to clinical practice seems difficult because BNP and NT-proBNP add only minor predictive value to clinical information. Shibazaki K. et al. [100] concluded that plasma BNP > 100.0 pg/mL (OR, 3.94; 95% confidence intervals (CI), 2.31–6.73, *p* < 0.001) were found to be independently associated with long-term mortality. Sayan S. and Kotan D. [106] concluded that plasma BNP levels were increased in the acute phase of stroke and can be used as a biomarker for mortality. Li J. et al. [103] and Bunevicius A. et al. [107] identified that the level of NT-proBNP was positively correlated with the National Institutes of Health Stroke Scale score and stroke severity, respectively.

### 2.2. Correlation with Cardioembolic Stroke

Ischemic stroke is subclassified into different etiologies, which includes atherosclerosis, cardioembolic, lacunar, others [109], and undetermined cases [110]. They differ in their underlying mechanisms behind the stroke event [111,112,113]. The most commonly used classification scheme is the Trial of Org 10,172 in Acute Stroke Treatment (TOAST) classification [114,115,116,117]. Stroke etiology classification’s main value is for therapeutic decision-making in order to reduce the time to IV-tPA admission [109]. The average time is 3 h from stroke symptoms, and it was found to be useful only when administered within 4.5 h [18,19]. However, ischemic stroke etiologies’ classification schemes are complex, time-consuming (between hours to days), and require professional personnel. Therefore, over the past years, different biomarkers have been examined for use in ischemic stroke etiology classification [49]. Several recent studies concluded that BNP and NT-proBNP elevated serum levels show a correlation with cardioembolic stroke [118,119,120,121,122,123,124,125,126,127,128,129,130,131,132,133,134,135,136,137,138,139,140]. In addition, BNP and NT-proBNP showed correlation with an atrial fibrillation (AF) condition, abnormal heart rhythm (arrhythmia), that was found to be associated with cardioembolic stroke. In AF, the source of increased plasma BNP could be due to an enlarged atrium [141]. Llombart V. et al. [119] reported that the blood levels of both BNP and NT-proBNP are significantly higher in cardioembolic stroke up to 72 h after symptoms onset, with a sensitivity > 90% and specificity > 80%. Yang H.-l. et al. [118] reported that the cardioembolic subgroup analysis showed that NT-proBNP had a slightly higher specificity and better capability for exclusion diagnosis. Kawase S. et al. [120] examined the correlations between plasma BNP level and conventional risk factors for ischemic stroke. The results showed that the mean plasma level of BNP was significantly higher for cardioembolic (366.6 pg/mL) than for non-cardioembolic (105.6 pg/mL; *p* < 0.01).

Moreover, in a study by Kara K. et al. [126], BNP distinguished the incidence of cardioembolic stroke. Chaudhuri J. R. et al. [122] investigated the association of plasma BNP levels in acute ischemic stroke subtypes and their outcomes. The results showed that among the stroke subtypes, elevated BNP levels were observed in 75% of cardioembolic stroke patients, 45.8% of small artery disease patients, 43.1% of larger artery atherosclerosis patients, and 34.5% of stroke of undetermined etiology patients. They concluded that an elevated BNP level is an independent marker for cardioembolic stroke and poor outcome at 90 days follow-up after the stroke incident. The same conclusion was received in a study by Cojocaru I. M. et al. [123], which showed that the level of plasma proBNP may be useful in distinguishing cardioembolic stroke from other stroke subtypes. Moreover, Bai J. et al. [133] reported that BNP showed a summary sensitivity of 0.65 (95% CI: 0.63–0.68) and a summary specificity of 0.85 (95% CI: 0.83–0.87). NT-proBNP showed a summary sensitivity of 0.55 (95% CI: 0.52–0.59) and a summary specificity of 0.93 (95% CI: 0.91–0.94). Nakamura M. et al. [135] reported that the ability of BNP to predict the incidence of cardioembolic stroke was excellent (area under the curve (AUC)-receiver operating characteristic (ROC) = 0.81). Also, when BNP was added to other well-known risk factors, the ability to predict cardioembolic stroke significantly improved: 4-year follow-up, *p* = 0.018; 8-year follow-up, *p* = 0.009; net reclassification improvement = 0.968, *p* < 0.0001: integrated discrimination improvement = 0.039, *p* < 0.05.

Strengthening these findings, Wu Z. et al. [121] examined the use of a POC test platform for plasma BNP detection in preliminary recognition of cardioembolic stroke patients in the ED. The results showed that the mean BNP concentration was significantly higher in the cardioembolic group than in the other 3 stroke subtypes: (1) large artery atherosclerosis; (2) small artery occlusion (e.g., lacunar); and (3) stroke of other determined etiology or stroke of other undetermined etiology (*p* < 0.01). The plasma BNP level greater than 66.5 pg/mL had good corresponding diagnostic performance in the preliminary recognition of cardioembolic stroke patients, with a sensitivity of 75.56% and a specificity of 87.40%. Their conclusion was that a plasma BNP level greater than 66.5 pg/mL as a reference index had good corresponding diagnostic performance in the preliminary recognition of cardioembolic stroke patients. However, the single BNP biomarker cannot be used individually to confidently identify the cardioembolic subtype as a diagnosis. In another study by Wu Z. et al. [124], the plasma BNP concentration was measured immediately at the bedside. The target was to recognize the patients with cardioembolic stroke as soon as possible due to high risks and poor long-term outcomes, including mortality risk. They concluded that the BNP testing at bedside upon admission could be suggested as an addition to early stroke management guidelines as a strategy for improving stroke subtype classification, predicting the development of atrial fibrillation after admission, and risk stratification.

### 2.3. Indication on Second Stroke Recurrence

BNP plasma and NT-proBNP serum levels can also indicate second stroke recurrence [142,143,144,145]. Shibazaki K. et al. [142] investigated whether BNP levels could be used as a biomarker to predict recurrent stroke in ischemic stroke survivors. Consecutive patients within 24 h of ischemic stroke symptom onset were prospectively enrolled and admission plasma BNP levels were measured. Survivors were followed for up to 12 months after stroke onset. A total of 793 patients who were alive at hospital discharge included 42 (5%) patients who had a recurrent stroke. There were no differences in BNP levels between the two groups. With respect to 257 patients with AF, BNP levels were significantly higher in the recurrence group than in the non-recurrence group (426.0 vs. 192.0 pg/mL, *p* = 0.0007). The BNP optimal cutoff level was >300.0 pg/mL, with 80% sensitivity and 73% specificity to distinguish the recurrence group from the non-recurrence group in stroke patients with AF. In another study by the same group, Shibazaki K. et al. [143] examined whether BNP levels are associated with early recurrent stroke in cardioembolic stroke patients. Admission plasma BNP levels were measured. Recurrent stroke was identified as the occurrence of additional neurologic deficits and the appearance of a new infarct on neuroimaging. The results showed that 17 patients (5%) had a recurrent stroke during hospitalization. The median interval time from stroke symptom onset to the recurrent stroke event was 4 days (range, 0–30). BNP levels were significantly higher in the recurrence group than in the non-recurrence group (304.1 vs. 206.5 pg/mL, *p* = 0.029). The BNP optimal cutoff level was >255.0 pg/mL, with 76% sensitivity and 60% specificity to distinguish the recurrence group from the non-recurrence group. They concluded that plasma BNP can be a useful biomarker for predicting early recurrent stroke events during hospitalization in cardioembolic stroke patients. A similar conclusion was also observed in a study by Mortezabeigi H. R. et al. [144], which concluded that BNP is capable of predicting TIA recurrence. Rodríguez-Castro E. et al. [145] reported that a cut-off point of 800 pg/mL of NT-proBNP predicted stroke with a sensitivity of 64% and a specificity of 79% (*p* < 0.001), and was independently associated with a higher risk of stroke after a TIA (OR: 6.65, *p* < 0.001).

**Table 1:** B-type natriuretic peptide association in stroke pathology.

Stroke Pathology	B-Type Natriuretic Peptide Level	Classification Performance	Study	Ref.
Increased Mortality	BNP = 118.2 vs. 60.9 pg/mL; *p* < 0.001)	BNP > 65.3 pg/mL; OR = 1.97; *p* = 0.034)	Montaner J. et al., 2012	[102]
	NT-proBNP levels in the deceased group = 3280 pg/mL	Significantly (*p* < 0.001) higher than in the survival group = 926.30 pg/mL	Chen X. et al., 2012	[101]
	BNP/NT-proBNP = 255.78 pg/mL (95% CI 105.10–406.47, *p* = 0.001)	BNP = OR 2.30, 95% CI 1.32–4.01 and NT-proBNP OR = 2.63, 95% CI 1.75–3.94	García-Berrocoso T. et al., 2013	[97]
	BNP > 100.0 pg/mL	BNP OR = 3.94, 95% CI, 2.31–6.73, *p* < 0.001	Shibazaki K. et al., 2013	[100]
	BNP = 284.16 ± 382.79 pg/mL at presentation and 273.78 ± 451.91 pg/mL at 72 h	BNP = 25.29 ± 13.47 pg/mL in healthy individuals as control group	Sayan S. and Kotan D. 2016	[106]
Cardioembolic Etiology	BNP = OR 15.8 (95% CI: 9.92–25.20)	Sensitivity = 78% (95% CI: 71%–87%) and specificity = 83% (95% CI: 77%–87%)	Yang H.-l. et al., 2014	[118]
	BNP = 366.6 pg/mL in cardioembolic patients	Non-cardioembolic = 105.6 pg/mL; *p* < 0.01)	Kawase S. et al., 2015	[120]
	BNP > 66.5 pg/mL	Sensitivity of 75.56% and a specificity of 87.40%	Wu Z. et al., 2015	[121]
	Elevated BNP levels were observed in 75% of cardioembolic stroke patients	Elevated BNP levels were observed in 45.8% of small artery disease patients, 43.1% of larger artery atherosclerosis patients and 34.5% of stroke of undetermined etiology	Chaudhuri J. R. et al., 2015	[122]
	BNP AUC-ROC = 0.81	Net reclassification improvement = 0.968, *p* < 0.0001, integrated discrimination improvement = 0.039, *p* < 0.05	Nakamura M. et al., 2018	[135]
Stroke Recurrence	BNP > 300.0 pg/mL	Sensitivity = 80% and specificity = 73%	Shibazaki K. et al., 2014	[142]
	BNP > 255.0 pg/mL	Sensitivity = 76% and specificity = 60%	Shibazaki K. et al., 2014	[143]
	NT-proBNP > 800.0 pg/mL	Sensitivity = 64% and specificity = 79%	Rodríguez-Castro E. et al., 2020	[145]

Abbreviations: 95% confidence interval (95% CI); odds ratio (OR); area under the curve (AUC); receiver operating characteristic (ROC).

## 3. Detection of B-Type Natriuretic Peptides

### 3.1. Point-of-Care Biosensor Platform

**Figure 3:**
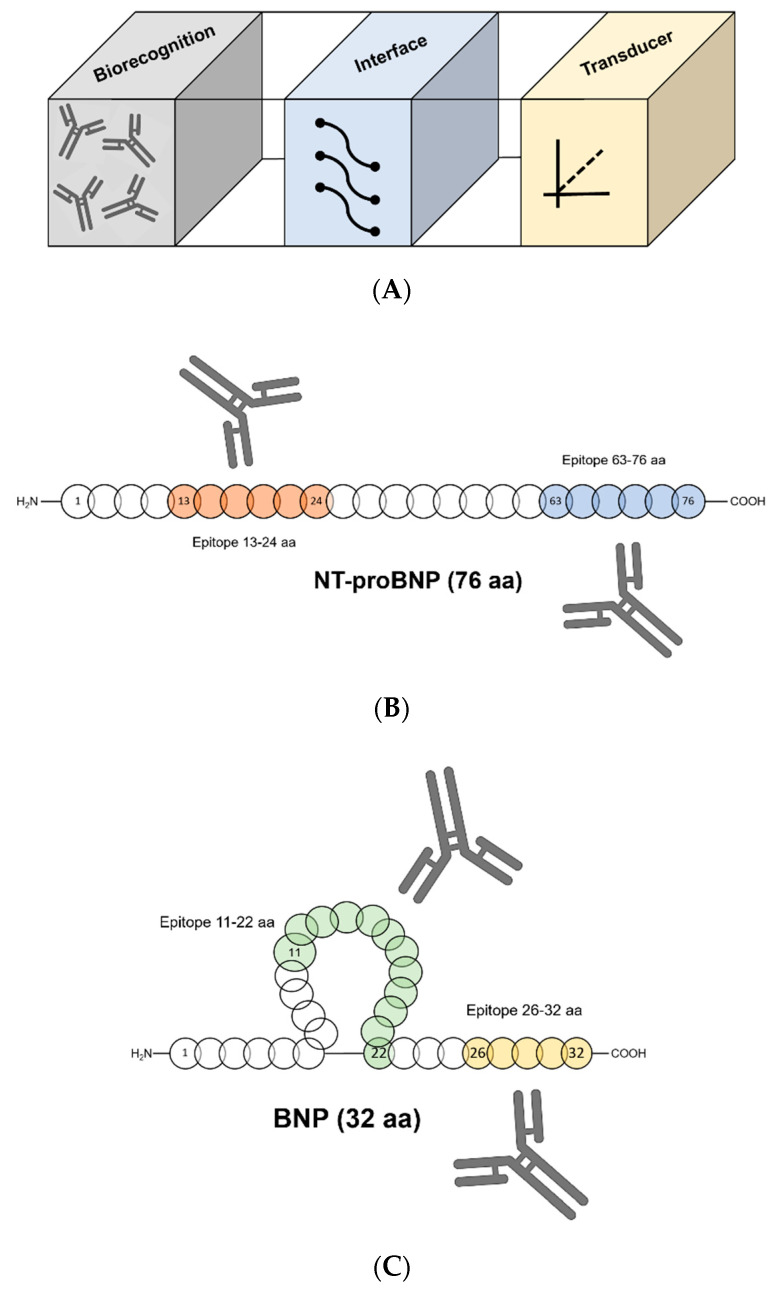
Detection of B-type natriuretic peptide. (**A**) Point-of-care biosensor platform; (**B**) N-terminal pro-B-type natriuretic peptide (NT-proBNP) epitopes (13–24 aa and 63–76 aa); (**C**) B-type natriuretic peptide (BNP) epitopes (11–22 aa and 26–32 aa).

A POC biosensor platform is a rapid test that obtains results within minutes, which is user-friendly, robust, and can be used on-site [146]. Successful examples include the glucometer [147] and lateral flow pregnancy test. These assay platform advantages include mobility, fast data processing, simple measurement, and the small volume of the sample required [148]. The enzyme-linked immunosorbent assay (ELISA) is the most common technology for immunoassay [149], with high sensitivity but complicated with multiple steps. A POC biosensor platform can have sufficient sensitivity while enabling a more practical approach [150,151]. It is an integrated biorecognition-transducer device, capable of giving quantitative results [152,153,154]. It is based on: (1) biorecognition, which allows the detection of target biological molecule; (2) the interface, the system’s main structure and function, and (3) the transducer that enables signal measurement and result processing (Figure 3). The choice of the biorecognition element (e.g., antibodies) is made according to the target analyte. Towards the creation of POC assays for commercial applications in healthcare diagnostics, there are constant efforts to minimize the size of the assay and still to obtain sensitive and accurate analyte detection, as well as to simplify the fabrication process [155,156,157]. There is an unmet need for customized POC in stroke care [158,159,160].

### 3.2. Attractive Epitopes for The Detection of NT-proBNP

The sensing of b-type natriuretic peptides for clinically useful diagnostic assays demonstrates analytical problems, mainly with circulating NT-proBNP [161], assay specificity, and analyte stability [162,163,164,165]. Several previous studies examined the development of NT-proBNP immunoassays [166,167,168]. There is no consensus on the exact circulating peptides that are derived from proBNP [80,169,170]. In addition, it was reported that proBNP undergo O-linked glycosylation [89], which further interferes with antibody recognition [171]. Moreover, molecular heterogeneity was also reported to influence NT-proBNP measurement [85,172]. Ala-Kopsala M. et al. examined the use of mass spectrometry to determine that even when there is heterogeneity in circulating NT-proBNP, still some specific epitopes of the peptide show higher stability, and therefore are more attractive for immunoassay development [161]. Two of those peptides were identified as NT-proBNP1–36 and NT-proBNP1–62/64; the masking of the NT-proBNP mid-region epitopes is likely due to oligomers that are formed. Most of the antibodies that are used in NT-proBNP immunoassays are directed against its terminal parts [85,173,174,175,176,177]. However, evidence was found that the N-terminus of NT-proBNP is susceptible to modifications that occur in the blood and alter its immune-reactivity [161,163]. These modifications may be the reason behind the large variance of circulating NT-proBNP concentrations that are reported by several immunoassays that are directed to its N-terminus [162,164,178,179]. Additionally, Seferian K. R. et al. examined endogenous NT-proBNP by applying multiple immunochemical approaches by using a panel of monoclonal antibodies (MABs) that are specific to different epitopes of the NT-proBNP peptide [171]. It was reported that the C-terminus of NT-proBNP is vulnerable to proteolysis, while the epitopes in its mid-fragment may be concealed by glycosylation. As a result, the mid-fragment of the NT-proBNP peptide is almost invisible for antibody recognition. It was concluded that MAbs specific to the N- and C-terminal parts of NT-proBNP (epitopes 13–24 and 63–76) are the best candidates to be used in an optimal assay for NT-proBNP detection (Figure 3). This conclusion contrasts with the conclusion accepted in the studies that were previously presented, which prioritized the recognition of the central part of the NT-proBNP molecule in order to avoid the blood-modification vulnerability of the non-mid-region epitopes. For example, NT-proBNP is often measured via a sandwich assay. However, NT-proBNP contains seven sites for O-linked glycosylation. Therefore, it is possible that one antibody in the sandwich assay may recognize one of the glycosylation sites. As a result, the serum NT-proBNP levels that are measured in such assay systems might be less accurate. Special attention should be paid to epitope profiling in the design of NT-proBNP assays.

### 3.3. Sandwich Immunoassay Formats for the Detection of BNP

The sensing of BNP is mostly conducted in a sandwich immunoassay format. The majority of the BNP sandwich immunoassays show the use of 2 monoclonal antibodies (MABs) that are specific for different BNP epitopes. One of the MABs is specific for the BNP peptide ring structure and the other MAB is specific for the C terminus or the N terminus of the BNP peptide. However, as previously discussed, both of these binding sites are susceptible to modifications in the blood that also have an influence on their immune reactivity. A study by Tamm N. N. et al. [180] shows the development of a novel BNP and proBNP immunoassay, which is based on a new form of a single epitope sandwich immunoassay. This immunoassay requires only a short BNP11–22 fragment (FGRKMDRISSSS) for the detection (Figure 3). The capture antibody (Ab) recognizes the BNP epitope, whereas the detection antibody is specific to the immune complex of the capture antibody and the BNP peptide (Ab-BNP). It was concluded that the developed single epitope sandwich immunoassay recognized both BNP and proBNP with the same efficiency and sensitivity than conventional sandwich BNP immunoassays. In addition, it demonstrated less susceptibility to antigen degradation. Also, this assay still maintained a short detection time and a broad linearity range (0.00023–17.6 nmol/L). Moreover, the developed assay was compared with two commercial assays that measure BNP for HF. The first, ARCHITECT^®^ BNP Assay Performance Verification, AACC 2006, uses a capture antibody from Scios (anti-BNP 106.3) that recognizes the ring structure and possibly part of the arm extending to the C terminus, and a detection antibody (anti-BNP BC203) from Shionogi that is specific to the C terminus of the molecule. The second, 50E1-24C5 HyTest in-house BNP assay uses similar epitope specificity of the antibodies. These two monoclonal antibodies, 106.3 and BC203 demonstrate high affinities to BNP and bind two distant epitopes [181].

### 3.4. Commercial Immunossays

BNP is a well-known diagnostic and prognostic biomarker in congestive HF [182]. Over the past decades, several commercial measurement devices for BNP detection in HF were developed (Table 2) [183]. In the literature, there are earlier studies that showed the measurement of BNP in three commercial assays: TRIAGE (fully automated immunoassay), IRMA (non-competitive immune-radiometric assay), and RIA (competitive immune-radiometric assay). Del Ry S. et al. [184] compared TRIAGE with IRMA. The TRIAGE method is a non-competitive immune-fluorometric sandwich assay which uses two different binding phases that are specific for two different epitopes of the BNP amino acid chain. A polyclonal antibody is included in the fluorescent immunoassay reagents, which are contained in the assay device, and a monoclonal antibody is immobilized in the detection lane. The mean reading time of the TRIAGE method was 14.5 ± 8.6 min. The TRIAGE method is used in emergency units, where usually only a few samples must be measured in a short time. The IRMA method is mostly preferred for pathophysiological studies which require the highest degree of precision and sensitivity for simultaneous measurement of several stored plasma samples or tissue extracts. A previous study by Clerico A. et al. [185] compared IRMA with RIA. The IRMA method is based on the solid-phase sandwich system, which uses two monoclonal antibodies prepared against two sterically remote epitopes of the peptide molecule. The first antibody is coated on the beads solid-phase and the second was radio-labeled. The IRMA method showed better sensitivity and a wider working range sensitivity (about 2 ng/L) than those of RIA methods. Moreover, the normal range found with these methods was similar to the one generally reported by using the most accurate methods. It was concluded that the IRMA method is preferable for the measurement of plasma BNP for experimental studies and as a routine assay because it is more practical, sensitive, and accurate than RIA procedures.

In addition, Fellner S. et al. [186] evaluated a new POC platform, responss^®^IQ. This assay is an immunoassay platform utilizing evanescent field total internal reflection fluorescence (TIRF) detection and active microfluidics controlled by optical sensors. A BNP assay was developed based on this system. The device consists of a single-use cartridge, which contains all the required biomaterials, as well as an instrument that moves the liquid and controls the microfluidic assay steps with the aid of optical sensors and measures the TIRF assay. In this assay, the limit of detection (LOD) achieved was 2.3 ± 1 pg/mL BNP. Ishida J. et al. [187] compared the analytical performance of two single-step measurement POC devices for BNP measurement. The two compared devices were a small-footprint immune-chromatography reader of BNP (Rapidpia^®^) and the commercially available SHIONOSPOT^®^ Reader as the index. The Rapidpia^®^ BNP assay demonstrated correlations between whole blood and plasma samples between those with the index SHIONOSPOT^®^ Reader, y = 0.93x + 0.88, R^2^ = 0.98 and y = 1.08x − 6.67, R^2^ = 0.93, respectively. Based on the reported findings, the two POC assays showed comparable results. The main objective of the Rapidpia^®^ test device is to measure BNP rapidly at the patient’s home or in the ambulance [188]. In the same study [187], three additional devices were mentioned which are commercially available for the measurement of BNP in Japan: MI02^®^ (Shionogi & Co., Ltd., Osaka, Japan), AIA^®^ (Tosoh Co., Ltd., Tokyo, Japan), and CL-JACK^®^ (Kyowa Medex Co., Ltd., Tokyo, Japan). Additionally, a technology report conducted by Oxford University reviewed POC testing for BNP measurement [189]. Several POC BNP testing devices were identified and they either measure BNP or NT-proBNP. The additional reported devices are as follow: (1) BNP measurement: the Alere Heart Check System (Alere, Stockport, UK). It is a handheld POC sensor that detects BNP from a 15 μL blood sample that is obtained by a finger prick. The results are obtained in 15 min. (2) NT-proBNP measurement: the RAMP 200 Clinical System (Response Biomedical, Vancouver, BC, Canada; no UK distributor identified) CE marked. The sensor measures NT-proBNP from an ethylenediaminetetraacetic acid (EDTA) whole blood sample, and the results are obtained in 15 min. The sensor has a reported lower LOD of 18 pg/mL and an upper limit of linearity of 23,450 pg/mL. The POC sensor weighs approximately 2 kg and is portable. (3) NT-proBNP measurement: the Cobas h232/Cardiac Reader (Roche Diagnostics, Burgess Hill, UK). It is a handheld sensor POC device that detects NT-proBNP from a 150 μL sample of heparinized venous blood. The results are obtained in 12 min. The sensor POC device has a reported lower and upper LOD of 60 pg/mL and 3000 pg/mL, respectively.

## 4. Conclusions

The hidden information and technological advancements behind the utilization of biomarkers for stroke triaging are significant. Stroke biomarkers can revolutionize the way that stroke patients are diagnosed, monitored, and how they recover. Different biomarkers indicate different cascades and exhibit unique expression patterns which are connected to certain pathologies in the human body. Over the past decades, BNP and its derivative NT-proBNP are increasingly investigated and highlighted as significant cardiovascular biomarkers. Previous studies reported on the usefulness of BNP and NT-proBNP for stroke triaging. They showed an association with increased mortality in stroke, correlation with cardioembolic stroke, and indication of second stroke recurrence. Recent scientific efforts have also been conducted for the technological advancement of a bedside POC device for BNP and NT-proBNP measurements. The conclusions discussed in this review may hopefully assist the major efforts that are currently being conducted in order to improve the care for stroke patients. An acronyms summary is presented in Table 3.

## Figures and Tables

**Table 2:** Food and Drug Administration (FDA) approved B-type natriuretic peptide immunoassays.

510(K) Number	Product	Company	First Approval Date	Update Approval Date
K873133	BNP-AMYLASE TEST	SCLAVO INC.		10/6/1987
K032335	MAS CARDIOIMMUNE PROBNP	MEDICAL ANALYSIS SYSTEMS INC.		8/19/2003
K033606	ABBOTT AXSYM B-TYPE NATRIURETIC PEPTIDE (BNP) MICROPARTICLE ENZYME IMMUNOASSAY (MEIA) TEST	AXIS-SHIELD DIAGNOSTICS LTD.		1/30/2004
K043584	LIQUICHEK BNP CONTROL	BIO-RAD LABORATORIES INC.		2/8/2005
K051265	ADVIA IMMUNO MODULAR SYSTEM (IMS) B-TYPE NATRIURETIC PEPTIDE (BNP) ASSAY	BAYER HEALTHCARE LLC	6/23/2003	6/13/2005
K052789	TRIAGE BNP TEST FOR BECKMAN COULTER IMMUNOASSAY SYSTEMS MODEL 98200	BIOSITE INCORPORATED	2/29/2000	1/23/2006
K051596	STATUS FIRST CHF (CONGESTIVE HEART FAILURE) NT-PROBNP MODEL 20204	NANOGEN INC.		3/13/2006
K060964	ARCHITECT BNP ASSAY MODEL 8K28	FUJIREBIO DIAGNOSTICS INC.		5/25/2006
K053597	I-STAT B-TYPE NATRIURETIC PEPTIDE (BNP)	I-STAT CORPORATION		7/21/2006
K071834	STRATUS CS ACUTE CARE NT-PROBNP TESTPAK MODEL CPBNPM	DADE BEHRING INC.	2/15/2005	8/17/2007
K072189	PATHFAST NTPROBNP AND D-DIMER TESTS	MITSUBISHI KAGAKU IATRON		2/5/2008
K073091	VIDAS NT-PROBNP ASSAY MODEL 30449	BIOMERIEUX INC.		2/29/2008
K080578	DIMENSION VISTA N-TERMINAL PRO-BRAIN NATRIURETIC PEPTIDE (PBNP) FLEX REAGENT CARTRIDGE (K6423A) DIMENSION VISTA	SIEMENS HEALTHCARE DIAGNOSTICS INC.	7/20/2004	5/16/2008
K063662	RAMP NT-PROBNP ASSAY	RESPONSE BIOMEDICAL CORP.		7/21/2008
K092649	ELECSYS PROBNP II STAT IMMUNOASSAY AND ELECSYS PROBNP II CALSET MODELS 05390109-160 04842472-190	ROCHE DIAGNOSTICS CORP.	11/19/2002	2/4/2010

**Table 3:** Acronyms summary.

Number	Acronym	Full Term
1	Ab	Antibody
2	AF	Atrial Fibrillation
3	ANP	Atrial Natriuretic Peptide
4	AUC	Area Under the Curve
5	BNP	B-type Natriuretic Peptide
6	CI	Confidence Interval
7	CT	Computed Tomography
8	ED	Emergency Department
9	EDTA	Ethylenediaminetetraacetic Acid
10	ELISA	Enzyme-Linked Immunosorbent Assay
11	FDA	Food and Drug Administration
12	HF	Heart Failure
13	LOD	Limit Of Detection
14	LTC	Long-Term Care
15	MAB	Monoclonal Antibody
16	MRI	Magnetic Resonance Imaging
17	NIHSS	National Institutes of Health Stroke Scale
18	NT-proBNP	N-Terminal pro-B-type Natriuretic Peptide
19	OR	Odds Ratio
20	POC	Point-Of-Care
21	ROC	Receiver Operating Characteristic
22	TIA	Transient Ischemic Attack
23	TOAST	Trial of Org 10,172 in Acute Stroke Treatment
24	tPA	Tissue Plasminogen Activator
